# Hidradenitis suppurativa: an update on connecting the tracts

**DOI:** 10.12688/f1000research.11337.1

**Published:** 2017-07-28

**Authors:** Mallory K Smith, Cynthia L Nicholson, Angela Parks-Miller, Iltefat H Hamzavi

**Affiliations:** 1 Wayne State University School of Medicine, Detroit, MI, USA; 2 Henry Ford Hospital Department of Dermatology, Detroit, MI, USA

**Keywords:** hidradenitis suppurativa, treatment, comorbidities, pathophysiology, mental health, sexual health, update

## Abstract

Hidradenitis suppurativa (HS) is a devastating disease involving abscesses, sinus tracts, and inflammation classically affecting the axilla, groin, and/or anogenital region. Although the disease pathogenesis is not fully understood, recent advances suggest that HS pathology runs much deeper than the cutaneous manifestations. It is now believed that HS is a systemic inflammatory disease that gives rise to the characteristic cutaneous manifestations. This disease is problematic for both patients and physicians to manage because of a variety of diagnostic and management difficulties. This article seeks to provide updates on the current understanding of HS to increase awareness and improve management.

## Introduction

Hidradenitis suppurativa (HS) is an inflammatory disease characterized by recurrent, painful abscesses and fistulous tracts ^1^. Patients with HS objectively have one of the lowest quality of life measures of any dermatologic disease ^2, 3^. Lesions characteristically occur in the axillary, groin, inframammary, and/or anogenital regions of the body, although other sites may be involved ^4^. HS lesions may progress to form sinus tracts and expansive abscesses. Sequelae include significant pain, scarring, and psychological distress ^5^. This article provides a comprehensive update on the current understanding of HS while also expanding upon the 2014 publication “Update on hidradenitis suppurativa: connecting the tracts” by Gill *et al.* ^1^.

## Background

### Epidemiology

HS was first described in 1839 by Velpeau in France ^1^. Since that time, and specifically throughout the past 10–20 years, much has been learned about this disease.

The average age of onset is during the early 20s, although pediatric cases are not uncommon. HS is more common in females than in males, at a rate of approximately 3:1. Additionally, HS more commonly occurs in the African-American population ^6– 9^. A recent retrospective study discovered significantly elevated rates of HS in African-American patients compared to Caucasian patients (6.4% to 3.9%, respectively), although further evidence is needed to support this association ^9^.

The prevalence of HS is a controversial topic, reported between 0.00033 and 4.1%, although underdiagnosis or improper diagnosis is common ^10^. A study from 1996 by Jemec *et al*. assessed 507 individuals undergoing sexually transmitted disease screening and calculated a point prevalence of 4.1% and a 1-year prevalence of 1.0% ^11^. In 2008, Revuz *et al*. carried out two case-control studies to explore HS prevalence, which was determined to be 1% ^12^. In 2013, the prevalence of HS in a health insured-only population (n = 7,927) was found to be 0.053% ^13^. In continuation, in 2014, a population-based study in Olmsted County, Minnesota, reviewed medical records from 1968–2008 and reported a prevalence of 0.13%. This study also indicated that the prevalence of HS is increasing, with rates of 4.3 per 100,000 between 1970 and 1979 to 9.6 per 100,000 between 2000 and 2008 ^14^. This study estimated a lower prevalence, although these findings are limited because this population’s racial and ethnic distribution was not representative of the United States. Another cross-sectional population study in Denmark evaluated HS demographics, and, overall, inverse recurrent suppuration (probable HS) prevalence was 2.1% based on a self-administered questionnaire given to adults aged 30–89 ^15^.

Overall, HS prevalence varies significantly based on study methodology; however, the disease appears to be more common than was previously considered.

### Exacerbating factors

Exacerbating factors of HS include, but are not limited to, mechanical stressors such as friction from clothing, sweating, and shaving ^16^. Of interest is the role of diet in the exacerbation or progression of disease. Owing to the possible role of hormonal regulation in disease pathogenesis, dairy was studied, as it may elevate insulin and promote androgen stimulation through the theorized hormonal effects of casein, whey, simple sugars, and 5-α reduced dihydrotestosterone (DHT). Forty-seven patients were placed on a dairy-free diet, and 83% clinically improved, while none experienced worsening of disease, compared to the control population that did not follow the dairy-free diet ^17^. This study reported impressive improvement; however, it failed to utilize validated assessment methods, and recall and non-response bias may have contributed to the outcome.

Brewer’s yeast is another dietary component of interest. One study followed 12 patients who eliminated foods containing brewer’s yeast, such as breads, beer, black tea, fermented cheese, and more, after the completion of HS surgery. Stabilization and eventual regression of HS throughout the one-year study period was reported ^18^. However, because of the small sample size, future studies are needed to further elucidate the role of brewer’s yeast in HS management.

In the authors’ experience, some patients report dietary triggers, while others report that strict adherence to various diets results in no change in symptoms, suggesting that further evidence is needed to evaluate this relationship. Nonetheless, the role of diet in HS disease management is a topic of great interest in both the general and the scientific communities.

## Associations and comorbidities

Obesity and cigarette smoking have known associations with HS ^12, 19^. A recent retrospective questionnaire reported that the prevalence of HS in an obese population was 18.1%, significantly higher than the general population. Additionally, 35% of these subjects reported improvement in HS symptoms following bariatric surgery and subsequent weight loss ^8^. Miller *et al*. observed body composition and basal metabolic rate in a cross-sectional study and determined that hospitalized HS patients had significantly higher body fat, visceral fat, BMI, waist circumference, and waist/hip ratio, while muscle and bone mass percentages were significantly lower than in controls ^20^. Additional comorbidities have been elucidated based on a case-control analysis of 2,292 HS patients, which found that arthropathies, obesity, dyslipidemia, hormonal disease such as polycystic ovarian syndrome, psychiatric disorders, drug or alcohol dependence, cardiovascular disease, and thyroid aberrancies occur more commonly in those with HS ^21^.

HS is also associated with metabolic syndrome, a multifaceted disease state including diabetes mellitus/insulin resistance, hypertension, dyslipidemia, and obesity. The prevalence of metabolic syndrome in HS patients was significantly higher than in controls in a retrospective chart review, at a rate of 50.6% compared to 30.2%, respectively ^22^. Other studies have supported this association, specifically regarding increased prevalence of obesity, hypertriglyceridemia, and insulin resistance in HS patients ^22, 23^.

Metabolic disease increases the risk of cardiovascular disease, a major health concern because of the associated increases in morbidity and mortality ^22^. Patients with HS are significantly more likely to have atherosclerotic disease than are controls, particularly carotid artery atherosclerosis, as determined through ultrasonography. Although causation was not determined, it was theorized that systemic inflammation associated with HS promoted atherosclerosis ^24^. This finding is concerning because cardiovascular disease is often subclinical and asymptomatic, with potentially deadly consequences. It has also been hypothesized that elevated levels of the inflammatory molecules interleukin (IL)-17 and tumor necrosis factor (TNF)-α contribute to endothelial damage. This provides immunologic context to relate cardiovascular disease to HS ^25^. The risk of cardiovascular events in patients with HS was also assessed in a Danish population-based cohort study of 5,964 subjects using data collected between 1997 and 2011. Compared to controls, HS patients were at an increased risk for ischemic stroke, cardiovascular-related mechanisms of death, major adverse cardiac events, and, surprisingly, all-cause mortality ^26^. These studies provide valuable knowledge for healthcare providers in all fields of medicine, which should guide preventative screening and interventions, along with public health research.

Chronic inflammatory diseases, such as inflammatory bowel disease (IBD), are also associated with HS ^27, 28^. The prevalence of IBD in 7,732 HS patients was 0.8%, significantly higher than the reported prevalence of IBD in the general population, at 0.3%. Additionally, the risk of new-onset IBD was significantly elevated in HS patients, specifically Crohn’s disease (CD), with a hazard risk of 2.19, and ulcerative colitis (UC), with a hazard risk of 1.63 ^29^. Another recent multicenter cross-sectional study indicated that the prevalence of IBD with HS was 3.3%; of that, 2.5% was CD and 0.8% was UC. Compared to the prevalence of IBD in the general Northern European population (0.41–0.74%), the prevalence of IBD was much higher in HS patients ^30^. Another recent population-based cohort study in Minnesota observed the rates of HS in 679 patients with IBD. The 10-year incidence of HS was 0.85%, and the 30-year incidence was 1.55%. Overall, patients with IBD were nine times more likely to develop HS compared to the general population ^31^. CD exhibits many parallels with HS, as both are chronic epithelial inflammatory diseases with inhabitation of bacterial flora, have a genetic predisposition, and respond to similar medication classes ^28^.

Owing to the chronic inflammation inflicted by recurrent HS lesions, squamous cell carcinoma (SCC) is a rare but serious complication of longstanding disease, with an incidence of 0.5–4.6% over the course of the disease ^27, 32^. It is hypothesized that free radical production and subsequent DNA damage provides the appropriate environment for malignant transformation, particularly in perianal regions of HS involvement ^33, 34^. It is unknown if systemic inflammation from HS itself contributes to the increased risk. Of great concern, high-risk human papilloma virus (HPV) type 16 has been isolated in pathology specimens of SCC lesions in HS patients ^35^. These associations are important for healthcare providers, as HPV testing and biopsy of a suspicious lesion with subsequent early intervention is associated with improved prognosis. Risk of malignancy is also an important consideration when beginning a biologic medication, which is associated with immunosuppression.

Psychiatric diseases, specifically anxiety and depression, are associated with HS, and this is discussed in further detail in the “Psychosocial impact” section ^36, 37^. Another notable association with HS is low socioeconomic status, as determined by a recent cross-sectional study by Deckers *et al*. The socioeconomic status of over 1,000 patients with HS was significantly lower than that of over 2,000 matched dermatologic controls. However, it is difficult to draw conclusions if downward drift is contributing to the socioeconomic status, as there are numerous confounding factors ^38^.

## Pathophysiology

The pathophysiology of HS is not fully understood, although it is thought to be multifactorial. The disease is now believed to be a systemic inflammatory condition, contrary to previous hypotheses classifying HS as a purely cutaneous disorder.

### Genetics

Both familial and sporadic cases of HS have been reported. It is thought that approximately 33% of HS cases are familial. Genetic variants of HS are heterogeneous, with several mutations identified throughout the genome, lacking a consistent genotype to phenotype correlation ^39– 41^. One study of 53 Chinese patients hypothesized to have familial HS were found to have a unique phenotype consisting of an increased male prevalence, earlier onset, and more severe disease involving atypical regions such as the neck and back ^42^. Because of the phenotypic variance in HS, Canoui-Poitrine *et al*. attempted to develop distinct phenotype-based subtypes of the disease, including axillary-mammary, follicular, and gluteal types. However, genetic analyses have not yet correlated with this grouping technique ^43^. Additionally, the specific phenotype “hidradenitis suppurativa fulminans” has been proposed to describe a specific, severe presentation of the disease ^40^. Phenotypic categorization will assist in future studies and determining a greater understanding of the varying presentations and genetics of this disease.

Some cases of HS are associated with syndromes, including pyoderma gangrenosum, acne, and suppurative hidradenitis (PASH) syndrome, related to mutations in proline-serine-threonine-phosphatase protein 1 (PSTPIP1). This gene is involved in the regulation of the inflammasome complex, a series of proteins and signaling pathways the body utilizes in response to inflammation-inducing stimuli ^44^. Another similar syndrome, pyogenic arthritis, pyoderma gangrenosum, acne, and hidradenitis suppurativa (PAPASH) syndrome, is also associated with PSTPIP1 gene mutations ^45^. Similarly, synovitis, acne, pustulosis, hyperostosis, and osteitis (SAPHO) syndrome has been associated with cases of HS. These relationships between HS and other auto-inflammatory diseases have bolstered the theory that HS may be auto-inflammatory in nature ^46^.

There is also evidence to support a relationship between familial HS and loss-of-function mutations in γ-secretase proteins, such as presenilin-1, presenilin enhancer-2, anterior pharynx defective 1, and nicastrin (NCSTN). These mutations were noted in variants of HS involving atypical regions such as the thighs and trunk ^7, 47^. The protease γ-secretase plays a role in the regulation of the canonical Notch signaling pathway, which is relevant to HS because Notch functions in the development of hair follicles, and as an immunomodulator in T-cell-mediated cellular immune responses. Inherited or acquired alterations in Notch signaling may play a pivotal role in HS disease pathogenesis ^48^. Cigarette smoking has been shown to decrease the activity of the Notch signaling pathway, further strengthening this association. Of note, having one of the above mutations does not guarantee disease development or progression ^49^.

Further, Xiao *et al*. analyzed the gene encoding NCSTN ( *NCSTN*) in a human keratinocyte cell line and found that *NCSTN* knockdown cells exhibited impaired γ-secretase activity, leading to more rapid proliferation and a greater proportion of cells in the S-phase of the cell cycle. This study bolstered the theory that a deficiency in the NCSTN gene in familial forms of HS may play a role in abnormal keratinocyte growth and proliferation ^50^. Interestingly, Duchatelet *et al*. discovered a NCSTN mutation in a patient with PASH syndrome, interrelating genetic and auto-inflammatory hypotheses of the pathogenesis of HS ^51^.

Finally, a retrospective study by Deckers *et al*. evaluated trends in early onset HS, defined as disease onset before age 13. A family history of HS was a significant factor in the diagnosis of early onset HS as well as more widespread disease. There were no reported differences in comorbidities in patients with early onset disease compared to controls with normal-onset HS ^52^.

### Etiology

Many previously accepted models of HS pathogenesis are being challenged. It is now believed that HS is a systemic inflammatory disease of multifactorial basis, possibly due to auto-inflammation ^53^.

It was formerly hypothesized that the disease originated in apocrine-bearing locations of the body, such as the groin and anogenital and axillary regions. However, the inframammary region, neck, trunk, and thighs are frequently involved as well ^7^. These areas experience recurrent friction, perhaps supporting the role of mechanical stress in the development of disease. Additionally, it was previously theorized that the first step in HS disease progression was the occlusion of the follicular infundibulum due to hyperkeratosis of adjacent epithelium. This hyperkeratosis was thought to act as a nidus for secondary infection, which caused the subsequent massive local inflammation associated with classic HS lesions ^48, 54^. Although follicular occlusion is paramount in the manifestations of HS, the instigating mechanism of occlusion is controversial. It has been hypothesized that these inflammatory events are instead secondary to an underlying aberrant inflammatory state in patients with HS instead of the primary cause of the disease ^55^.

Although the driving force behind the development of HS lesions is unknown, recent studies have proposed various theories to describe the instigating pathology. One study observed the basement membrane of lesional and clinically uninvolved skin and found abnormal epithelial support and basement membrane surrounding the follicular junction in lesional tissue based on periodic acid-Schiff (PAS) histologic staining. These findings suggested that individuals with HS may have an underlying anatomic defect in the basement membrane predisposing to secondary infections ^56^. Blok *et al*. similarly evaluated perilesional biopsies in patients with HS compared to healthy controls. Contrary to the previous findings, this study found neither a significant difference in PAS staining nor a difference in many other basement membrane constituents between patients and controls. However, this study observed significant upregulation of integrins α6 and β4 (signaling molecules with adhesive properties) within the sebaceous gland of HS patients compared to controls. The role of these integrins in HS is unclear at this time ^57^.

Additionally, it is known that keratinocytes and neutrophils play a role in the secretion of pro-inflammatory molecules in HS ^58^. Hotz *et al*. proposed that abnormal keratinocyte function plays a role in HS development. Compared to controls, keratinocytes in HS lesions showed a diminished inflammatory response to muramyl dipeptide, a pathogen-associated molecular pattern (PAMP) inflammatory antigen ^59^. Keratinocyte malfunction further suggests structural and molecular abnormalities allowing for follicular occlusion and disease progression. Although the precise cause of the follicular occlusion remains debated, cellular markers and other immunologic sources of inflammation are important topics of discussion in the ongoing search to determine the etiology of HS.

In summary, overarching themes of inflammation and abnormal cellular activity appear to provide the appropriate environment for the progression to HS clinical features. It is probable that many factors allow for altered cellular barrier mechanisms and anomalous secretion of pro-inflammatory cytokines in the progression to classic HS symptoms ^48^.

### Inflammation in hidradenitis suppurativa

HS is a systemic inflammatory disease, and auto-inflammation is suggested to play a role in disease pathogenesis. This theory was bolstered by the association of comorbid autoimmune and inflammatory diseases, abnormal biochemical findings, and an infiltration of innate and adaptive immune cells within both lesional and perilesional skin before clinical manifestations of the disease arise ^60^. In particular, auto-inflammatory diseases (AIDs) are rare, hereditary, unprovoked systemic inflammatory diseases that classically occur in the absence of infection or autoantibodies. These disorders are thought to be due to altered regulation and signaling patterns in the innate immune system. As described above, HS has been associated with many AIDs ^53, 61, 62^.

The exact cytokine profile of HS has yet to be determined, although abnormal levels of several inflammatory cytokines have been observed in HS, with notable elevations in IL-1β, IL-10, IL-11, IL-17A, and CXCL9 (monokine induced interferon [IFN]-γ). Additionally, mRNA and protein quantities of TNF-α, IL-1β, and IL-10 were reportedly elevated in HS ^63– 65^. Interleukins are a part of the body’s natural response to stressors, secreted by innate and adaptive immune cells, each with its own specific role in immunomodulation. It is important to note that individual cytokine profiles differ between patients ^66^. While an in-depth discussion of the abnormal cytokine profile in HS is beyond the scope of this review, notable findings and common cytokine trends associated with HS will be expanded upon.

Increased activity of the pro-inflammatory IL-23/Th17 pathway has been implicated in many chronic inflammatory diseases, recently including HS. Studies have supported that IL-12 and IL-23 were expressed in large quantities by macrophages in HS lesional skin, along with the infiltration of IL-17-producing helper-T and CD4 ^+^ T cells ^65, 67, 68^. IL-17-producing cells were similarly found in lesional and perilesional skin in HS patients. The knowledge of IL-17 as an activator of keratinocytes and source of inflammatory modulators in HS has provided important insights into the disease process as well as potential management options ^25, 58, 69^. These findings have important implications in the management of HS as ustekinumab, a monoclonal antibody against IL-23 and IL-12, has demonstrated some efficacy for HS management ^70^.

IL-1β is another pro-inflammatory cytokine elevated in lesional HS tissue ^63^. This cytokine is well known as a pyrogen and leukocyte-activating factor, among numerous other functions. A controlled clinical trial by Tzanetakou *et al*. determined that HS disease activity and exacerbations were attenuated with anakinra therapy. Anakinra is an antagonist of the IL-1 receptor, making the IL-1 pathway a reasonable target to pursue for disease management. Additionally, during the course of treatment with anakinra, the treatment group also exhibited decreased levels of other pro-inflammatory markers such as IL-6, TNF, and IFN-γ compared to controls, further supporting the role of the IL-1 pathway in HS pathogenesis ^71^.

IL-6 is another pro-inflammatory molecule of interest. This cytokine is elevated in inflammatory diseases such as rheumatoid arthritis, CD, and HS. Levels of IL-6 were significantly elevated in lesional HS tissue compared to controls in a recent study by Xu *et al*. ^72^. Interestingly, increased levels of IL-6 and C-reactive protein (CRP) were found to confer a poor response to infliximab treatment, making these markers reasonable adjunctive assessments in determining appropriate management ^73^. Further studies are needed to fully elucidate the role of IL-6 and methods to appropriately manage patients using this biochemical pathway.

Increased levels of chemotactic agents such as B-lymphocyte chemoattractant (BLC), CCL3, CCL5, and IL-16 have also been observed with HS ^63, 74^. These cytokines similarly play a role in inflammation, particularly the acute-phase reaction ^60^. Interestingly, markers such as IL-2, IL-4, IL-5, and IFN-γ were found to be extremely low in perilesional HS skin ^63^.

TNF-α is secreted by both innate and adaptive immune cells and has been implicated in the disease process of many other inflammatory conditions, such as psoriasis and inflammatory bowel disease. These findings have been instrumental in the development of biologic medications and led to the first approved biologic medication for HS, adalimumab ^75^. TNF-α has been shown to be elevated in HS through numerous studies, indicating important involvement in the disease pathogenesis, and as an effective target for management ^64, 74^.

Of note was the finding that levels of IL-1β, TNF-α, and IL-10 correlated with increased severity of HS, further supporting these markers as suitable targets for therapy ^63, 65, 74^. Following this work, Hotz *et al*. observed increased levels of CD4 ^+^ T cells in patients with HS and increased levels of IL-17 and IFN-γ from the aforementioned T cells ^59^. Another study observed cytokine concentrations in purulent drainage obtained from HS lesions and found that, overall, pro-inflammatory cytokines such as TNF-α, IL-1β, IL-1α, and IL-17 were increased in addition to elevations in anti-inflammatory cytokines IL-10 and IL-1ra, although each patient exhibited a unique cytokine profile. This multifaceted study also provided evidence that peripheral blood monocytes in HS patients produced fewer cytokines and were less active in responding to stimulation than were controls, indicating systemic involvement in the disease ^66^.

Antimicrobial peptides (AMPs), such as β-defensin (BD) 1, BD2, BD3, and other S100 proteins, such as psoriasin and calgranulins A and B, are believed to play a role in the pathogenesis of HS. AMPs are natural defense molecules constitutively expressed by keratinocytes, although irritants and other inflammatory stimulators can modify their expression. Tissue samples from six HS patients exhibited overexpression of AMPs compared to skin from healthy controls ^76^. Interestingly, a similar study of seven patients evaluated AMPs and cytokines within HS lesions and found a relative decrease in all analyzed AMPs. It was thought that the decrease in AMPs contributed to the susceptibility to secondary infections. This study also provided evidence that a deficiency of IL-22 and IL-20 may be responsible for decreased AMP levels, although further studies are needed on this topic ^77^.

From a different molecular perspective, microRNAs (miRNAs) are short, non-coding nucleotide chains involved in gene regulation. miRNAs are thought to regulate inflammatory markers in many chronic inflammatory diseases, such as psoriasis. In 2016, Hessam *et al*. observed that the miRNA modulators Drosha and DGRC8 were significantly downregulated in perilesional HS tissue, although no change was observed in these markers in lesional tissue ^78^. Following this work, Hessam *et al*. evaluated levels of specific miRNA molecules. Interestingly, significant overexpression of several miRNAs was observed in lesional compared to healthy control skin, including miRNA-31 and miRNA-125b. Of note, miRNA-31 is thought to be involved in regulating skin inflammation, and miRNA-125b is proposed as an important regulator of keratinocyte proliferation and TNF-α production ^79^. Although further studies are needed to fully assess the dysregulation of miRNA in HS, these findings contributed to the understanding of the HS disease process at the molecular level.

This information, which will surely be supplemented by future studies, has provided the basis of knowledge for understanding HS as an inflammatory disease and supported the use of biologics in its management.

### Bacterial infection and biofilms

The normal bacterial flora count of the skin is approximately 1 trillion microorganisms existing in a symbiotic state with the human body. The skin hosts a variety of microbiomes, with each body part creating a specialized topographic niche for different microorganisms. Most exist in harmony with the body, although imbalances have been observed in disease processes ^80^. In HS, bacterial colonization develops, causing suppurating lesions, although the exact role of microorganisms in disease pathogenesis is unknown. The immune response to the bacteria is likely abnormal, resulting in difficult-to-treat lesions.

Most HS lesions are poly-microbial in nature. The most frequently cultured bacteria from HS lesions are Gram-positive cocci and rods, such as coagulase-negative *Staphlococci* and *Corynebacterium*, as well as various subspecies of anaerobic bacteria ^81^. One study of recalcitrant HS observed that the most commonly cultured organisms were *Corynebacterium* species (14.0%), *Staphylococcus epidermidis* (13.1%), and *Staphylococcus aureus* (10.4%) ^82^. Interestingly, Jahns *et al*. evaluated 27 samples of HS lesional tissue and did not find *S. aureus* in any of the assessed samples. Of note, this was a retrospective analysis of fixed tissue using immunofluorescent labeling of samples that were not controlled for lesional depth. The absence of *S. aureus* was thought to be due to the anaerobic nature within deep HS lesions, contributing to the understanding that the identification of bacterial species is likely lesional depth dependent ^83^. It is likely that sample depth and methods of organismal assessment played a role in this finding. Guet-Revillet carried out a prospective study using a series of bacterial cultures from 102 HS lesions and determined that *S. lugdunensis*, a normal aerobic member of the skin flora, was present in 58% of the samples, making it one of the most common pathogens observed ^84^. The pathogens most commonly implicated in HS inflammatory lesions are benign on unaffected skin. This indicates a structural or immunologic abnormality that allows for bacterial overgrowth in HS patients, although future studies are needed to validate this hypothesis. There is still much to be learned about the microbiome and its role in HS.

Of particular interest is the complication of bacterial biofilms in HS lesions. Biofilms are observed in a variety of diseases and are associated with classic findings of chronic, non-healing inflammatory lesions with episodic acute-on-chronic flares. Often responsive at first, then resistant to antibiotic treatments, biofilm-associated lesions cause a great deal of difficulty in management. Bacterial metabolic adaptations often lower the efficacy of antimicrobial agents, making the lesions more difficult to treat ^85^. Biofilm development also results in a physical barrier, further contributing to decreased antibiotic efficacy. The three strains of bacteria most commonly implicated in biofilm disorders are *Pseudomonas aeruginosa, Enterococcus faecalis,* and *S. aureus* ^86^.

A recent study evaluated perilesional biopsies of 42 patients with chronic HS and found that 67% of the samples showed evidence of biofilms. Larger biofilms were associated with more extensive disease ^85^. Interestingly, another study evaluated acute HS lesions in 10 patients and found no evidence of biofilms ^87^. Although the sample size was small, this finding supports the current understanding that biofilms develop in chronic lesions. The discovery of biofilms in HS has improved the understanding of the disease process and will lead to many new directions in treatment options targeting this aspect of pathology ^85, 88^. Methods of managing chronic wounds associated with biofilms are discussed below.

### Classification systems

A variety of classification systems have been proposed to assess disease severity, although, recently, many methods have been developed in an attempt to capture treatment efficacy.

Hurley staging was one of the first techniques to categorize HS severity based on lesion type and proximity of neighboring lesions ^1, 89^. Of clinical utility, a positive correlation of CRP levels was associated with more advanced Hurley staging ^90^. The Sartorius system incorporates lesion quantity and location, distance between lesions, and presence of unaffected skin in clinical staging ^5^. The Physician Global Assessment (PGA) scale details six stages of disease process based on clinical criteria, with stage-specific guidelines for management and follow-up ^4^.

More recently, the HS Clinical Response (HiSCR) was formulated to evaluate HS stability and assess clinical improvement. The HiSCR system first determines the patient’s baseline number of abscesses and inflammatory nodules (AN count). HiSCR is a clinically determined endpoint of the disease; achievement of HiSCR50 occurs when a 50% reduction in total AN count is observed, with no increase in abscesses or draining fistulas compared to baseline ^91^. The ability to assess the transient inflammatory components of HS is a strength of this assessment technique. Overall, although clinical staging has been helpful in the management of HS, scoring techniques often underestimate disease severity compared to other modalities, such as ultrasound ^92^.

The underestimation of severity through clinical diagnosis may lead to less desirable outcomes in management. Wortsman *et al*. reported a surprising 82% modification in management approach, from medical to surgical, owing to anatomic details observed on ultrasound that were missed on physical examination ^92^. The Sonographic Scoring of HS (SOS-HS) technique, developed by Wortsman *et al*., is a standardized method of HS severity assessment using ultrasound techniques to examine HS lesions ^92– 94^. The use of ultrasonography in HS staging may be a valuable tool to stage HS and guide management.

Overall, a consensus of diagnostic criteria and treatment recommendations is needed to improve and streamline HS management ^4^.

## Management of hidradenitis suppurativa

**Figure 1.**
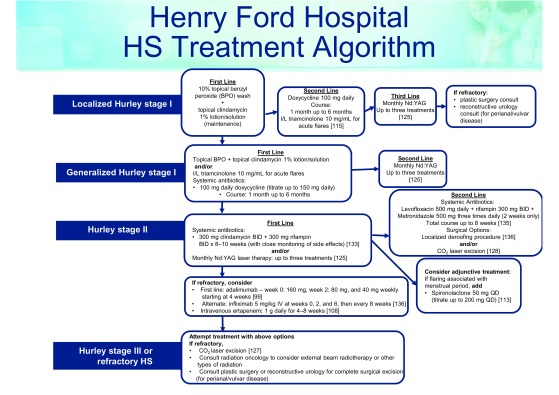
Henry Ford Hospital hidradenitis suppurativa (HS) treatment algorithm. This treatment algorithm is used in the Henry Ford Dermatology Clinic, which provides care to more than 1,000 patients with HS. The algorithm is based on a combination of evidence-based research and provider experience. BID, *bis in die*; I/L, intralesional; IV, intravenously; Nd:YAG, neodymium-doped yttrium aluminum garnet; QD, *quaque die*.

Treatment for HS is guided by disease severity and symptomatology. While the medical, surgical, and laser management sections are separated for ease of discussion, these treatment options are not exclusive but rather exist on a continuum and are often combined to optimize treatment response. Additionally, it is important to address and properly treat underlying medical conditions to comprehensively manage patients. To date, there is no widely accepted, evidence-based treatment algorithm, although evidence-based treatment ladders have been suggested. The Henry Ford Health System in Detroit, MI, proposed an algorithm of HS management based on the treatment of more than 1,000 patients in a dedicated follicular disorders clinic (see Figure 1).

### Medical management

Medical treatment optimizes a combination of agents including keratolytics, such as benzoyl peroxide or resorcinol, topical antibiotics, systemic antibiotics, hormonal modulators, systemic retinoids, intralesional corticosteroids, and, more recently, biologics and immunomodulators. The goal of medical management is to decrease disease burden and symptoms with the intent of reaching a state of remission.

Gulliver *et al*. used the European Dermatology Forum guidelines to create a treatment algorithm. This article recommended that a dermatologist well versed in HS should act as the primary coordinator of care and, initially, lifestyle modifications should always be offered. Treatment for HS begins with topical or oral antibiotics, such as clindamycin and oral tetracycline, respectively, followed by the use of rifampicin if there is no improvement. Next, biologic therapy is offered, which should be continued if the patient responds. If the response is inadequate or the patient cannot take biologics, transition to second- or third-line options is reasonable, including surgical or laser approaches when options have been exhausted, as discussed below. The treatment regimen of advanced HS is currently in a fluid state, although research and evidence-based medicine is guiding the way towards a consensus ^95^.

Antibiotic treatments for HS begin as broad-spectrum regimens. Bettoli *et al*. suggested that, to improve outcomes, a multidisciplinary team approach as well as antimicrobial stewardship should be implemented to prevent long-term sequelae such as resistance, relapses, and over- or under-treatment of disease ^96^. Despite our best efforts, unfortunately antimicrobial resistance in HS has been reported ^97^.

When topical medications and oral antibiotics fail, or the disease has progressed, biologic medications can be recommended. Adalimumab, a TNF-α inhibitor, has the most evidence supporting its use. Two open-label, phase II, randomized controlled trials (RCTs) determined that weekly dosing of adalimumab statistically improved clinical response when compared to placebo controls ^98, 99^. Following these studies, two phase III trials, PIONEER I and II, described statistically improved clinical response in the adalimumab treatment group compared to placebo after 12 weeks. Specifically, 41.8% of the treated group reached HiSCR compared to 26.0% of the placebo group in PIONEER I ( *p* = 0.003) and 58.9% versus 27.6% in PIONEER II ( *p*<0.001), respectively. PIONEER II also noted a statistically significant improvement in Sartorius score, from a mean of 81.4 before treatment to 52.5 at week 12 ( *p*<0.001) ^75^.

Second-line treatments are reasonable options in non-responders. These include infliximab (another TNF-α inhibitor) and anti-androgen therapy. Other methods of management, although fewer data exist to support their efficacy, include metformin, IL-1 receptor antagonists such as anakinra, anti-IL-17 agents, and IL-12/IL-23 pathway inhibitors such as ustekinumab.

Infliximab and etanercept have been used in the management of HS. Infliximab was one of the first TNF-α inhibitors evaluated for HS. Previous studies indicated success with infliximab treatments ^100, 101^. In an eight-week, phase II study of 20 patients with moderate-to-severe HS treated with infliximab, significant improvement in pain, clinical disease severity, and quality of life were reported compared to controls ^102^. Subsequently, Moriarty *et al*. optimized dosing regimens to every four weeks ^99^. Although support for the use of infliximab is strong, adalimumab offers ease of administration compared to infliximab ^103^. However, in the authors’ experience, infliximab is more efficacious in refractory patients, despite not having FDA approval for this indication.

Etanercept is another TNF-α inhibitor of interest in the management of HS. Although initial studies were promising, subsequent studies failed to demonstrate efficacy ^104, 105^.

Because of the involvement of the IL-12/Th1 and IL-23/Th17 pathways in the pathogenesis of HS, ustekinumab, a human monoclonal antibody that targets IL-12 and IL-23, was proposed as a potential modulator of HS. The first open-label study used 45 or 90 mg of ustekinumab at 0, 4, 16, and 28 weeks, which resulted in improved Sartorius scoring in 82% of 17 total patients ^70^.

Anakinra is an IL-1 receptor antagonist that has been implicated in the treatment of HS because of the noted elevated levels of this inflammatory cytokine and is also used in the management of AIDs owing to the role of IL-1 as a pro-inflammatory agent in this spectrum of diseases as well ^106^. Two studies have reported the efficacy of anakinra for HS; the first was an open-label study, in which five patients given 100 mg of subcutaneous anakinra showed improvement based on the modified Sartorius scoring system after 8 weeks ^107^. The second study supported these findings in Hurley stage II and III patients treated with anakinra for 12 weeks. It was found that disease activity and IFN-γ levels significantly decreased compared to in controls ^71, 107^. These studies provided evidence to support continued assessments of anakinra as a plausible future treatment option.

Another promising treatment for HS is ertapenem. In a pilot study evaluating the use of ertapenem in patients with severe HS, a six-week course of 1 g of intravenous (IV) ertapenem daily followed by a consolidation treatment resulted in improved Sartorius scores. Further improvement was noted during the consolidation treatments. Patients who were not able to or did not complete the treatment stopped improving and some returned to their baseline severe disease ^108^.

Of interest in the management of HS is the use of metformin. In a small clinical study, 25 patients with refractory HS were initiated on metformin for a period of 24 weeks. Following the course, there was a significant improvement in Sartorius scores. This study seemed to strengthen the association among metabolic syndrome, disease severity, and perhaps hormonal imbalances in HS, as metformin also has anti-androgen properties ^109^. Although these initial findings were promising, subsequent studies are needed ^110^. It is important to always recommend lifestyle modifications in treatment plans, as metabolic syndrome increases the risk for many other medical complications.

Colchicine, an anti-inflammatory medication, was previously discredited in having a role in HS management ^111^. However, a 2017 study by Armyra *et al*. indicated that colchicine might provide some clinical benefit in combination with tetracycline. This prospective series study treated all 20 patients with 100 mg minocycline daily in combination with 0.5 mg colchicine daily for 3 months. Using Hurley and Dermatology Life Quality Index (DLQI) scoring techniques, significant improvement in HS symptoms was observed ^112^.

Anti-androgen therapy has also been used in the treatment of HS. A small case series involving 20 women with mild- to moderate-severity HS were treated with spironolactone, an androgen receptor blocker, which showed improvements in disease activity. However, some of the patients were concurrently taking oral contraception, and five were taking minocycline ^113^. The side effects of the medication were reported to be minimal, providing evidence to support further studies on the efficacy of spironolactone. Again, comorbid disease states may be a confounding factor, and lifestyle modifications for overall health are key to appropriately managing HS patients.

Biofilms pose a difficult challenge in HS management. For successful treatment, it is necessary to break down the biofilm. There is evidence to support the use of biofilm-penetrating gels, which consist of a pH buffer, benzalkonium chloride surfactant, as well as antimicrobial activity, in other chronic non-HS-related wounds ^114^. Although this study was not specific to HS, it is implied that similar mechanisms may benefit chronic HS lesions. Inhibition of biofilm formation is another mechanism that may benefit HS patients. In the Lubbock chronic wound biofilm (LCWB) model, xylitol, salicylic acid, erythritol, and two novel Sanguitec gels, combined in semi-solid wound dressing formulations, inhibited biofilm formation from *P. aeruginosa*, *E. faecalis*, and *S. aureus* in the laboratory setting ^86^. Future directions in HS research must include HS disease-specific biofilm management techniques to improve wound care.

The treatment of disease through intralesional corticosteroids is a unique method of symptomatic management, but, previously, evidence was limited. Intralesional triamcinolone in 10 patients resulted in both physician- and patient-reported improvement. Significant reductions in physician-assessed levels of erythema, edema, suppuration, and size were noted on days 1 and 2 following injections, while patient-reported pain scores were also significantly improved. This study provided evidence to support intralesional corticosteroid injections, although it was limited by the small sample size and lack of control group ^115^. Additionally, the natural history of HS lesions with progression towards reduced inflammation, pain, and drainage complicates the interpretation of these results.

Lifestyle modifications are a major form of management that should be addressed and recommended to all patients. In particular, it is known that cigarette smoking impedes healing. In a retrospective cohort study, non-smokers were significantly more likely to exhibit improvements following first-line treatments compared to smoking counterparts ^116^. In addition to smoking cessation, weight loss of more than 15% is associated with a significant improvement in disease severity ^8, 117^. It is beneficial to consider lifestyle modifications in the management of all patients with HS, along with routine treatments.

### Surgical and laser management

When medical management is ineffective, surgery is often the next step for HS treatment. Surgical management generally involves excision of the lesional material with or without the removal of associated scar tissue. There are both localized and extensive surgical interventions. Although there is no consensus on the best approach, procedures are carried out based on disease severity and location, with the overall goal of removing lesional tissue and sparing healthy skin to optimize outcomes.

Many approaches exist to manage HS, including local destruction, incision and drainage (I&D), standard unroofing, and wide excision techniques ^118^. Local destruction is used to ablate HS lesions and may be carried out with electrosurgery, cryotherapy, or laser removal. I&D may be used for decompression in acute episodes of unbearable pain. However, I&D does not assist in the long-term resolution of disease, as the inflamed tissue remains and infection is almost certain to recur ^119^. Unroofing techniques are effective for both small and large lesional units and are carried out by opening the surface of all connected abscesses and tracts within an HS lesion. The contents are removed by curettage, often leaving the site open to heal by secondary intention ^120^. Unroofing techniques are preferred for Hurley stage I/II, whereas skin-tissue-saving excision with electrosurgical peeling (STEEP) is preferred for Hurley stage II/III. STEEP similarly removes diseased, fibrotic tissue via electrosurgical loop while sparing healthy skin to decrease sequelae following the surgical procedure ^121, 122^. Lesions that cannot be unroofed may be excised, which includes the removal of the entire diseased area up to the margins of normal-appearing subcutaneous tissue ^118^.

The best surgical approach for long-term outcomes is controversial and is dependent on the patient’s disease severity and location of the lesions. A recent systematic review and meta-analysis assessed for recurrence rates with varying methods of surgical management. The lowest rates of recurrence occurred following wide excision therapy when compared to local excision and deroofing procedures. This study also indicated that recurrence rates were lowest with skin grafts and skin flaps compared to primary closure, although this was limited by retrospective analysis and lack of randomization of closures ^123^. Wide excision followed by secondary intention healing was also shown to be functional and aesthetically acceptable to patients ^124^. Bias may exist in the discrepancy between recurrence rates and type of closure owing to the fact that primary closure is possible only with smaller wound sizes compared to larger excisions that simply cannot be closed by primary intention. Although this has not been formally assessed, the size of the wound may play a role.

The use of lasers in HS management has gained recent popularity. The 1064 nm neodymium-doped yttrium aluminum garnet (Nd:YAG) laser appears to be an effective, novel modality in HS management. After a series of treatments, two trials reported decreases in HS-associated inflammation, scarring, and fibrosis, indicating successful, selective photothermolysis to manage the disease ^125, 126^. It is best used in recalcitrant Hurley stage I and II patients.

Another type of laser used in the treatment of HS is the carbon dioxide (CO _2_) laser, which is used to excise HS lesions and ablate pathologic tissues. CO _2_ laser has been used since the late 1980s for HS, although it is now increasing in popularity. Laser excision followed by marsupialization has been shown to be effective for the management of persistent or late-stage cases of HS, with overall patient satisfaction in post-operative quality of life and pain measures ^127, 128^. Additionally, a retrospective study (n = 58) of CO _2_ laser evaporation techniques reported a 29% recurrence rate of disease within 12.2 months of the procedure (noted around the borders of treated regions), while 95% of patients reported some or great improvement in disease status ^129^. A reported complication of CO _2_ excision is scar contracture, restricted range of motion, and delayed wound healing. Nicholson *et al*. determined that fractional CO _2_ therapy could be a helpful adjunct in these cases ^130^. For further reading, see 131– 134.

## Psychosocial impact

HS can be a debilitating disease, physically and emotionally, with increased prevalence of depression and psychiatric disease noted in this patient population. Onderdijk *et al*. evaluated patient-reported levels of depression, as determined through the Major Depression Inventory (MDI) questionnaire, which indicated higher scores for patients with HS compared to controls. However, clinically defined depression was not statistically more frequent. DLQI scores were significantly higher in patients with HS, indicating greater daily impact of disease ^37^. A more recent cross-sectional study utilized a database of over 4 million patients, 3,207 with HS, and found a significant association between depression and anxiety ^36^. Most recently, Kouris *et al*. noted a significant association between HS and depression, anxiety, and social isolation, as well as lower levels of self-esteem, compared to controls ^135^. These studies reinforced the need for psychiatric evaluations as part of routine care for HS patients.

Due to the sensitive location of HS lesions, sexual health is often a concern for patients and their partners. A survey-based multicenter cross-sectional study of 916 HS patients reported decreased quality of life and overall sexual health as assessed by scoring techniques such as Female Sexual Function Index, International Index of Erectile Function, and DLQI ^2^. This was one of the first large-scale assessments published on the matter. In the authors’ experience, coordination with psychiatrists, psychologists, and sexual health therapists experienced in the impacts of chronic disease and chronic pain can be a valuable resource in the multidisciplinary approach to manage HS.

Social isolation and loneliness are detrimental to overall health. In a review of over 3,000 individuals, Holt-Lundstat *et al*. found an increased risk of mortality in subjects who reported feelings of loneliness and social isolation. Mortality risk was comparable to well-established risk factors, such as obesity. Subjects reporting loneliness saw a 26% increased risk of mortality, while those reporting social isolation saw a 29% increased risk of mortality ^136^. To combat the impacts of social isolation and loneliness, support groups can be a useful resource for both patients and providers.

There are a variety of support groups dedicated to HS, the majority of which are social media based, and a few in-person support groups. Regardless of the format, support groups offer patients the ability to interact with others facing similar circumstances and provide a sense of validation that only others with HS can provide. As with other chronic diseases, the impacts of HS are felt by caregivers, spouses, partners, family members, and friends. Participation in support groups should be considered not only for the patient but also for the caregiver.

In the author’s experience, a support group that involves medical providers hosts a successful and productive format. Those with HS have often had unpleasant experiences in medicine, having suffered through mismanagement of their care and a delay in diagnosis ^10^. Provider involvement can ensure that accurate information about HS is disseminated and discussed. Patients can provide feedback on treatments and also make their own suggestions for new treatment options. Additionally, a provider- and patient-driven format provides an avenue to rebuild trust and the opportunity to gain insight into disease impacts through an informal setting without clinical time constraints.

## Future directions

Great strides in the understanding of HS have been achieved in recent years. However, additional studies are needed to improve treatment options and patient outcomes moving forward. Future directions include increasing the awareness of HS in all fields of medicine to improve diagnoses and management, as well as continued treatment exploration, using updated knowledge on the pathogenesis of disease as the framework for future studies. With these collaborative goals in mind, multidisciplinary teams will continue to strive for success in overcoming this distressing disease.
